# Risk of diabetes mellitus with olanzapine compared to clozapine: A systematic review and meta‐analysis

**DOI:** 10.1002/pcn5.70215

**Published:** 2025-10-14

**Authors:** Keigo Onda, Rizal Ichwansyah, Shuken Boku

**Affiliations:** ^1^ Department of Psychiatry Niigata University School of Medical and Dental Sciences Niigata Japan; ^2^ Department of Physiology Niigata University School of Medical and Dental Sciences Niigata Japan

**Keywords:** clozapine, diabetes mellitus, meta‐analysis, olanzapine

## Abstract

**Aim:**

Olanzapine and clozapine are second‐generation antipsychotics commonly associated with metabolic side effects. In Japan, olanzapine is designated as “principally contraindicated” for patients with diabetes, while clozapine is “used with caution,” despite similar pharmacological profiles. This study compares the risk of new‐onset diabetes mellitus between olanzapine and clozapine using adjusted effect estimates from observational studies.

**Methods:**

We conducted a systematic review and meta‐analysis of observational studies published between 2000 and 2025 that reported diabetes‐related outcomes in patients treated with olanzapine or clozapine. Adjusted odds ratios (aORs) and hazard ratios (HRs) were synthesized using a random‐effects model. In the absence of direct comparative data, the Bucher method was employed for indirect comparisons using a shared comparator.

**Results:**

Sixteen studies were included. Meta‐analysis of seven HR‐based studies revealed a significantly lower risk of new‐onset diabetes in the olanzapine group compared to clozapine (pooled HR = 0.73, 95% confidence interval [CI]: 0.63–0.85). On the other hand, the odds ratio (OR)‐based analysis (nine studies) showed no significant difference (pooled OR = 0.85, 95% CI: 0.66–1.09). Sensitivity analysis with only aORs and subgroup analysis by follow‐up duration revealed no significant differences.

**Conclusion:**

This study provides preliminary evidence that olanzapine is associated with a lower or at least comparable risk of new‐onset diabetes relative to clozapine. Considering that olanzapine is more tightly regulated than clozapine in clinical practice in Japan, these findings indicate the need for further confirmatory studies and accumulation of robust evidence to inform potential regulatory reclassification.

## INTRODUCTION

Antipsychotic medications are widely used in the treatment of psychiatric disorders such as schizophrenia. Although they are effective in controlling acute symptoms, preventing relapse, and supporting functional stabilization, their long‐term use is often complicated by metabolic adverse effects, including weight gain, dyslipidemia, and diabetes mellitus.^1^, ^2^, ^3^ Among second‐generation antipsychotics (SGAs), agents such as olanzapine, clozapine, and quetiapine are classified as multi‐acting receptor‐targeted antipsychotics (MARTAs), which have high affinities not only for dopamine D₂ receptors but also for histamine H₁, serotonin 5‐HT₂C, and muscarinic receptors. These pharmacodynamic properties contribute to significant metabolic disturbances.^4^, ^5^, ^6^

Given the risk of metabolic dysfunction associated with olanzapine and clozapine,^7^, ^8^, ^9^ many countries label both drugs as “use with caution” for patients with preexisting diabetes.^10^ In Japan, however, the regulatory stance is markedly different: while clozapine is classified under “use with caution,”^11^ olanzapine and quetiapine are designated as “principally contraindicated”^12^ for patients with diabetes. These differences in labeling may cause confusion in clinical practice. For example, whereas clozapine treatment can often be continued with appropriate glycemic control following the onset of diabetes, olanzapine or quetiapine are generally discontinued immediately.

Whether this regulatory distinction appropriately reflects the overall pharmacological profiles of each antipsychotic remains uncertain. These regulatory decisions appear to have been based largely on case reports and data from spontaneous adverse event reporting systems, rather than on robust comparative evidence.^13^ While such systems, including the Japanese Adverse Drug Event Report (JADER) database, are valuable for signal detection, the lack of denominator data limits their utility in estimating incidence rates or comparative risk.^14^, ^15^ These limitations pose challenges in assessing the relative safety profiles of different antipsychotics based on such data alone. Moreover, the regulatory stance in Japan toward olanzapine and quetiapine—despite their pharmacological similarities to clozapine—is notably stricter than in other countries, possibly reflecting ethnic factors, such as the higher susceptibility to glucose dysregulation observed in Asian and Oceanian populations.^16^, ^17^, ^18^ Therefore, to inform regulatory decisions, a meta‐analysis synthesizing adjusted relative risk, accounting for confounding factors such as ethnicity, is both appropriate and warranted.

A recent study in a Japanese population demonstrated that both olanzapine and clozapine were significantly associated with progression from normoglycemia to hyperglycemia. The hazard ratio (HR) was 2.06 (95% confidence interval [CI]: 1.05–4.05) for olanzapine and 4.25 (95% CI: 1.56–11.60) for clozapine.^19^ Moreover, while several international meta‐analyses^20^, ^21^, ^22^, ^23^, ^24^ have investigated the metabolic risks of antipsychotics, most have been based on absolute risk estimates from incidence rates, which may underestimate the true comparative risk. In addition, clozapine was often excluded from these analyses due to the limited number of available cases, making it less likely to be included as a comparator in quantitative syntheses.

In the present study, we aimed to systematically evaluate the relative risk of new‐onset diabetes, with a particular focus on comparing olanzapine to clozapine, using available observational data. By synthesizing adjusted effect estimates and applying indirect comparison methods such as the Bucher approach, we sought to provide evidence to inform future regulatory and clinical decision‐making in Japan.

## METHODS

### Study design

This study presents a systematic review and meta‐analysis of observational studies comparing the risk of diabetes mellitus associated with olanzapine and clozapine. The review was conducted in accordance with the Preferred Reporting Items for Systematic Reviews and Meta‐Analyses (PRISMA) guidelines and was prospectively registered in PROSPERO (registration number: CRD420251088815; registered on July 6, 2025). No protocol was prepared for this review.

### Literature search

A comprehensive literature search was conducted in the following databases: PubMed, Web of Science, ScienceDirect, Cochrane Library, and Ichushi Web. The search covered studies published between January 1, 2000, and December 31, 2025, and was conducted in July 2025. The primary search query was (olanzapine OR quetiapine) AND clozapine, combined with outcome‐related terms such as “diabetes mellitus,” “ketoacidosis,” and “HbA1c.” Controlled vocabulary terms (e.g., MeSH or Emtree) were used where applicable. Reference lists of eligible studies were also manually reviewed.

Two independent investigators (K. Onda and R. Ichwansyah) conducted the search and screening. Discrepancies were resolved through discussion. Only human studies were included. No language restrictions were applied, except for PubMed, which was limited to English‐language publications. Case reports were excluded through both filters and manual review.

Duplicate records were removed using R (version 4.3.2), an open‐source environment for statistical computing and graphics^25^ via exact matching of article titles and author names. Additionally, a machine learning‐based tool (ChatGPT, OpenAI) was used as an auxiliary tool during abstract screening and preliminary classification (e.g., include/exclude/unclear). All classifications were subsequently confirmed by human investigators. The full list of records identified during the initial screening is provided as an additional file for review but not for publication.

### Eligibility criteria

#### Inclusion

Observational studies (e.g., cohort or nested case‐control) involving clozapine or olanzapine and reporting outcomes such as new‐onset diabetes, diabetic ketoacidosis (DKA), or diabetes‐related mortality. Comparators could include first‐generation antipsychotics (FGAs), other SGAs, or general population.

#### Exclusion

Non‐original studies (such as case reports, reviews, letters, or comments) and studies not reporting diabetes‐related outcomes.

### Data extraction and risk of bias assessment

Two investigators independently extracted data using a standardized form. Extracted variables included: author, year, study design, region, sample size, patient demographics, comparison groups, follow‐up duration, outcome measures, confounder adjustment, and reported effect sizes—HRs and odds ratios (ORs), each with a 95% CI.

Risk of bias was assessed using the Newcastle‐Ottawa Scale (NOS), examining selection bias, exposure/outcome ascertainment, and adjustment for confounders.

### Statistical analysis

Random‐effects meta‐analyses were conducted to calculate pooled effect estimates (HR or OR). Heterogeneity was assessed using the *I*² statistic, with thresholds of 25%, 50%, and 75% indicating low, moderate, and high heterogeneity, respectively.

Statistical analyses were conducted using R. The meta package^26^ was used for main analyses, and the metafor package^27^ for meta‐regression and subgroup analyses. Forest and funnel plots were generated to visualize effect sizes and assess potential publication bias.

### Analysis strategy

#### Primary analysis

We conducted a meta‐analysis based on observational studies to compare the risk of developing diabetes mellitus between olanzapine and clozapine. To account for major confounding factors, we prioritized adjusted effect estimates—specifically, HRs and adjusted odds ratios (aORs). Since only one study directly compared olanzapine and clozapine, we adopted the Bucher method^28^ for indirect comparisons as the primary analytical approach, using a common comparator, typically FGAs. Publication bias was evaluated using funnel plots, Egger's test, and Begg's test.

The Bucher method, which calculates the log‐transformed effect size and standard error (SE) was applied as follows:

$$\log\left({\mathrm{RR}}_{\left\{\mathrm{AB}\right\}}\right)=\log\left({\mathrm{RR}}_{\left\{\mathrm{AC}\right\}}\right)-\log\left({\mathrm{RR}}_{\left\{\mathrm{BC}\right\}}\right)$$

$$\mathrm{SE}(\log\left({\mathrm{RR}}_{\left\{\mathrm{AB}\right\}}\right))=\sqrt{\left\{{\mathrm{SE}}^{2\left(\log({\mathrm{RR}}_{\left\{\mathrm{AC}\right\}}\right))}+{\mathrm{SE}}^{2\left(\log({\mathrm{RR}}_{\left\{\mathrm{BC}\right\}}\right))}\right\}}$$

where:

- RR denotes a relative risk measure, such as HR or OR,
- ${\mathrm{RR}}_{\left\{\mathrm{AB}\right\}}$ is the indirectly estimated effect between Treatments A and B (e.g., olanzapine vs. clozapine),
- ${\mathrm{RR}}_{\left\{\mathrm{AC}\right\}}$ and ${\mathrm{RR}}_{\left\{\mathrm{BC}\right\}}$ represent effect estimates for Treatments A and B compared to a common Comparator C (e.g., FGAs), and
- SE is the standard error of the log‐transformed effect measure.

#### Sensitivity and subgroup analyses

Sensitivity analyses were conducted by excluding studies that reported only unadjusted ORs to assess the robustness of the results. Subgroup analyses were conducted based on follow‐up duration.

## RESULTS

### Study selection

**Figure 1:**
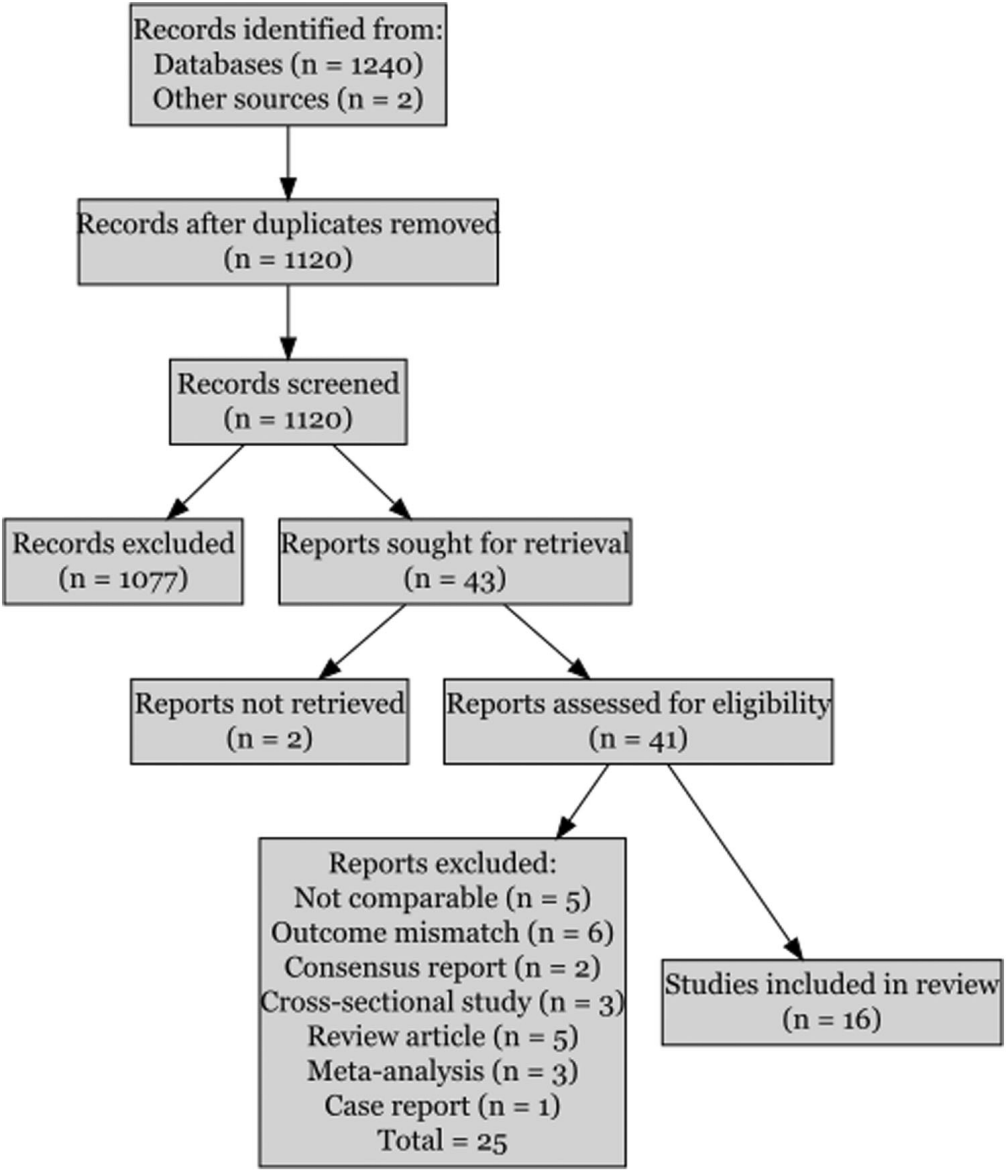
Preferred Reporting Items for Systematic Reviews and Meta‐Analyses (PRISMA) flow diagram summarizing the study selection process.

A total of 1244 records were identified, of which 1120 remained after duplicate removal. Following title and abstract screening, 43 articles were retrieved for full‐text review, and 16 studies met the eligibility criteria and were included in the meta‐analysis. The detailed screening process is illustrated in the PRISMA flow diagram (Figure 1).

### Study characteristics

The 16 included studies were published between 2002 and 2013 and employed observational designs, primarily retrospective cohort and nested case‐control studies. Sample sizes ranged from 50 to over 30,000 participants. Most studies reported adjusted effect estimates, including HR from Cox proportional hazards models and aOR derived from multivariable logistic regression that accounted for covariates such as age, gender, region, and comorbidities. These adjusted estimates were prioritized to improve the accuracy of the association between antipsychotic exposure and diabetes risk.

Outcomes of interest included new‐onset diabetes and, in one study, diabetes onset with DKA. Diagnostic categories included schizophrenia (most commonly defined by ICD codes) and, in some studies, bipolar disorder, while diabetes was defined using ICD codes, antidiabetic prescriptions, or the American Diabetes Association (ADA) guidelines. Detailed study characteristics are presented in Table 1 and Supporting Information S3: Table 1.

**Table 1:** Characteristics of included studies.

ID	First author	Year	Country	Study design	Sample size	Outcome	Effect size type	Effect size (95% CI)	Risk of bias (NOS)
30	Buse	2003	United States	Retrospective cohort	14,140	Diabetes (prescription)	HR	0.83 (0.37–1.88)	7/9
31	Yood	2009	United States	Retrospective cohort	17,266	Diabetes (ICD‐9 code)	HR	0.66 (0.18–2.42)	8/9
32	Lambert	2006	United States	Nested case‐control	21,073	Diabetes (ICD‐9 code)	HR	0.76 (0.53–1.09)	8/9
33	Guo	2007	United States	Nested case‐control	54	Diabetes (ICD‐9 code)	HR	1.28 (0.36–4.49)	8/9
34	Miller	2005	United States	Retrospective cohort	2070	Diabetes (ICD‐9 code)	HR	0.77 (0.34–1.76)	8/9
35	Leslie	2004	United States	Retrospective cohort	56,849	Diabetes (prescription)	HR	0.73 (0.60–0.89)	8/9
36	Ollendorf	2004	United States	Retrospective cohort	972	Diabetes (ICD‐9 code)	HR	0.68 (0.51–1.03)	8/9
37	Gianfrancesco	2006	United States	Retrospective cohort	31,465	Diabetes (ICD‐9 code)	aOR	0.77 (0.57–1.04)	8/9
38	Lambert	2005	United States	Matched case‐control	879	Diabetes (ICD‐9 code)	aOR	1.00 (0.82–1.22)	8/9
39	Rubio	2005	Spain	Case‐control	996	Diabetes (ADA criteria)	aOR	0.77 (0.25–2.36)	8/9
40	Gianfrancesco	2002	United States	Retrospective cohort	1099	Diabetes (ICD‐9 code)	aOR	0.42 (0.08–2.21)	8/9
41	Moisan	2013	Canada	Nested case‐control	10,339	Diabetes (ICD‐9 code)	aOR	1.27 (0.68–2.39)	9/9
42	Nielsen	2010	Denmark	Prospective cohort	1751	Diabetes (ICD‐10 code)	aOR	0.68 (0.41–1.12)	9/9
43	Winkel	2008	Belgium	Prospective cohort	71	Diabetes (ADA criteria)	OR	0.75 (0.15–3.87)	6/9
44	Henderson	2007	United States	Retrospective cohort	1002	Diabetes with DKA or HHS	OR	0.34 (0.11–1.07)	6/9
45	Feng	2012	Sweden	Retrospective cohort	50	Diabetes (ADA criteria)	OR	17.20 (0.92–319.91)	8/9

*Note*: Summary of observational studies comparing the risk of new‐onset diabetes mellitus between olanzapine and clozapine. Full details of study setting, data source, duration, analysis, and covariates are provided in Supporting Information S3: Table 1.

Abbreviations: aOR, adjusted odds ratio; CI, confidence interval; HR, hazard ratio; NOS, Newcastle‐Ottawa Scale; OR, odds ratio.

### Risk of bias within studies

Risk of bias was assessed using the NOS. The majority of studies (87.5%) were rated as having low risk of bias, while the remainder were of moderate quality due to limitations such as a lack of multivariate adjustment.

### Comparative risk of new‐onset diabetes mellitus

#### Olanzapine versus clozapine (HR‐based analysis)

**Figure 2:**
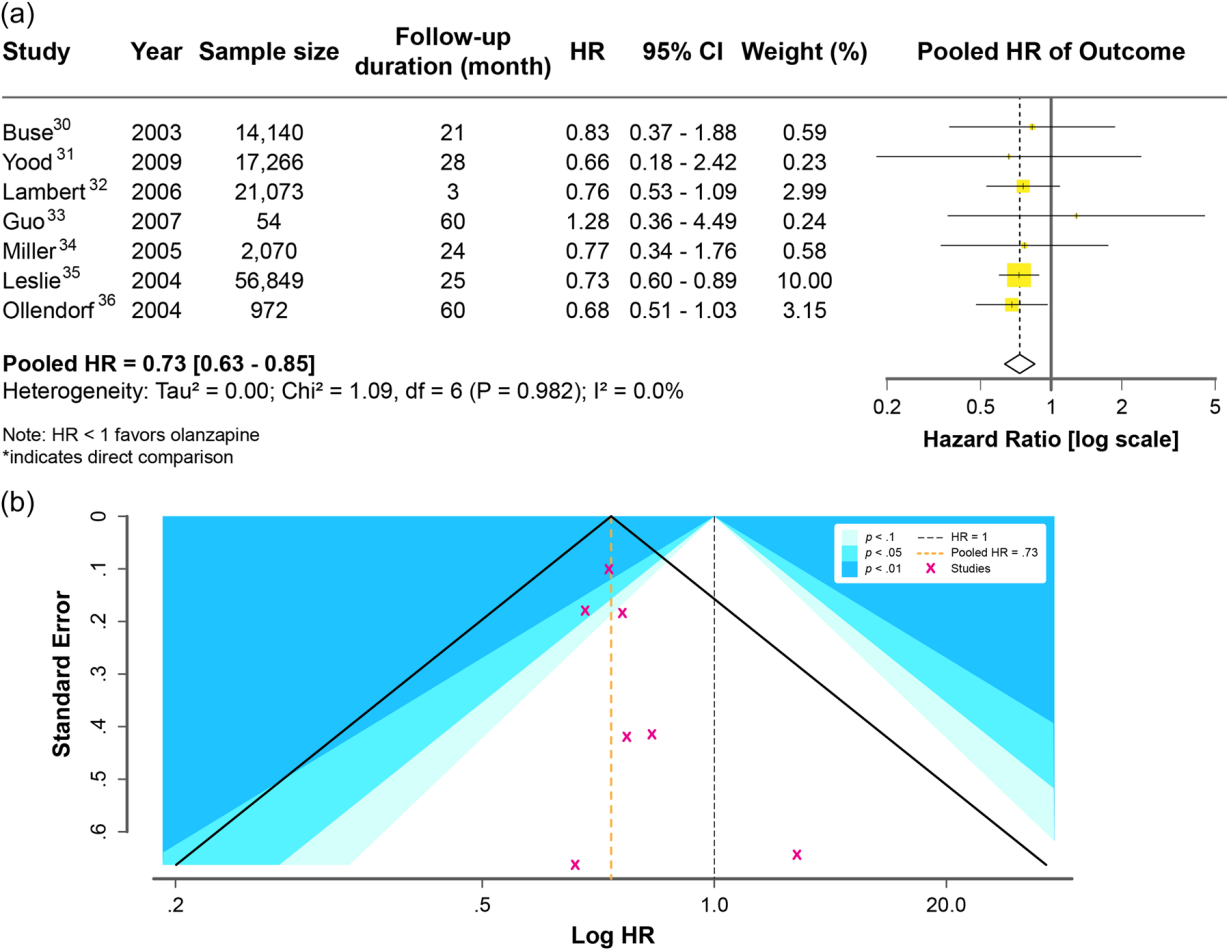
(a) Forest plot of hazard ratios for diabetes risk (log‐scaled *x*‐axis). CI, confidence interval; HR, hazard ratio. (b) Funnel plot with contour shading for significance regions (*p* < 0.1, 0.05, 0.01).

Seven studies^29^, ^30^, ^31^, ^32^, ^33^, ^34^, ^35^ reporting HRs were meta‐analyzed. The pooled HR was 0.73 (95% CI: 0.63–0.85), suggesting a significantly lower diabetes incidence in the olanzapine group (Figure 2a). There was no observed heterogeneity (*I*² = 0.0%, *p* = 0.98).

Notably, only the study by Ollendorf et al. directly compared olanzapine with clozapine. The remaining studies employed indirect comparisons, where HRs for each drug were estimated separately within broader observational cohorts using a common reference group.

To assess potential publication bias, a contour‐enhanced funnel plot was generated (Figure 2b). Visual inspection did not reveal marked asymmetry. Egger's test showed no significant small‐study effects (*t* = 1.30, df = 5, *p* = 0.25; bias estimate = 0.35, SE = 0.27), and Begg's test was also nonsignificant (*z* = 0.75, *p* = 0.45; bias estimate = 5.00, SE = 6.66). However, due to the limited number of studies (*n* = 7), the statistical power to detect publication bias was low.

#### Olanzapine versus clozapine (OR‐based analysis)

**Figure 3:**
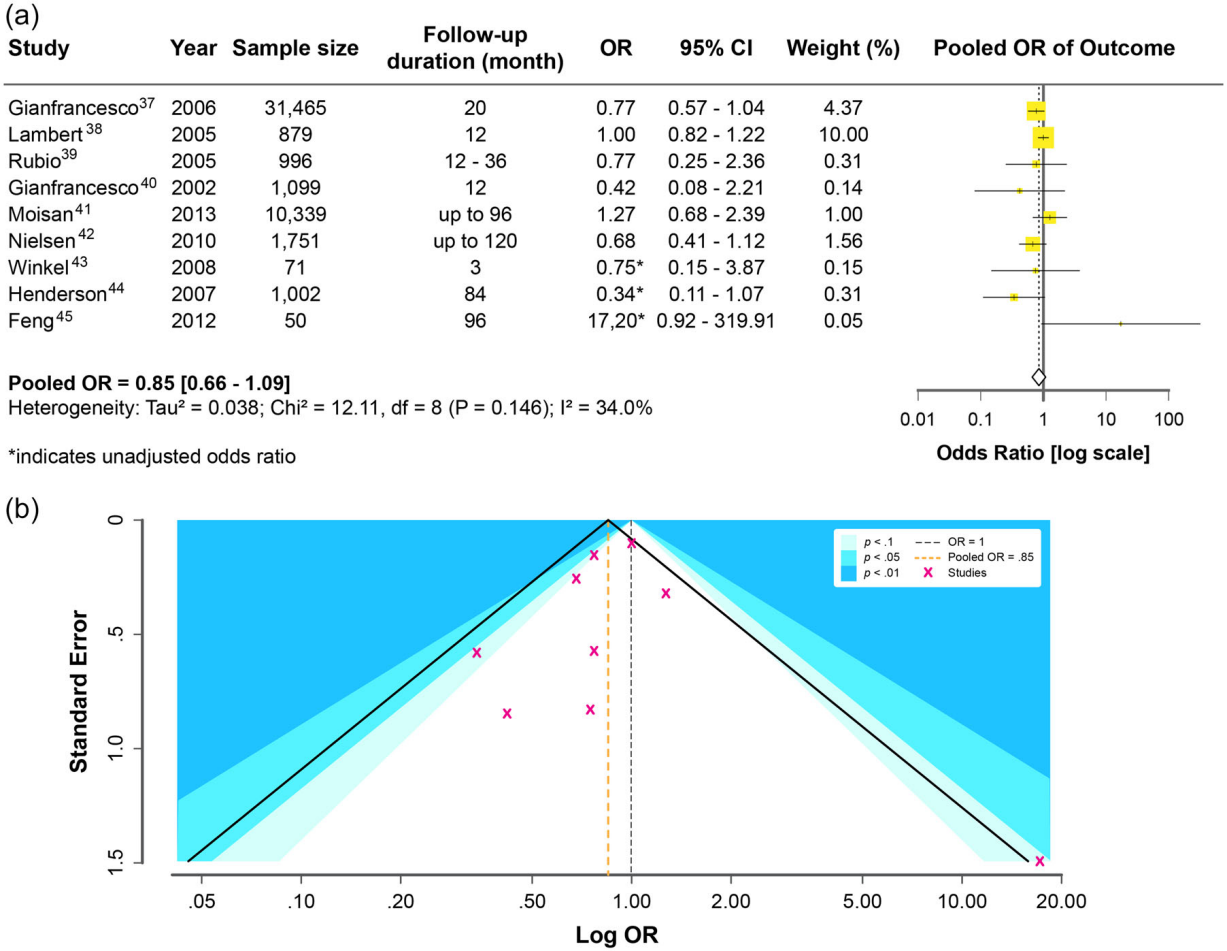
(a) Forest plot of odds ratios for diabetes risk (log‐scaled *x*‐axis). CI, confidence interval; OR, odds ratio. (b) Funnel plot with contour shading for significant regions (*p* < 0.1, 0.05, 0.01).

A total of nine studies^36^, ^37^, ^38^, ^39^, ^40^, ^41^, ^42^, ^43^, ^44^ were included in the OR‐based meta‐analysis, incorporating both adjusted and unadjusted ORs. The pooled OR was 0.85 (95% CI: 0.66–1.09), indicating no statistically significant difference in diabetes risk between the two antipsychotics (Figure 3a). Moderate heterogeneity was observed (*I*² = 34.0%, *p* = 0.15). Several studies contributed minimal weight due to wide CIs or small sample sizes.

The funnel plot appeared symmetric (Figure 3b). Egger's test showed no significant evidence of publication bias (*t* = –0.38, *p* = 0.71), and Begg's test was also nonsignificant (*z* = 0.42, *p* = 0.68).

#### Sensitivity and subgroup analyses

To complement the main analysis, a sensitivity analysis was conducted by excluding unadjusted estimates. This secondary meta‐analysis included aORs from studies that accounted for key confounders and yielded a pooled aOR of 0.90 (95% CI: 0.76–1.06), with minimal heterogeneity (*I*² = 6.4%) (Supporting Information S1: Figure 1).

Additionally, a subgroup analysis based on follow‐up duration (≤24 months vs. >24 months) was conducted. The pooled ORs were 0.91 (95% CI: 0.77–1.07) for the short‐term group and 0.87 (95% CI: 0.40–1.90) for the long‐term group. The test for subgroup differences was not statistically significant (*p* = 0.92) (Supporting Information S2: Figure 2), suggesting that follow‐up duration did not meaningfully affect the relative risk of diabetes between the two drugs.

## DISCUSSION

Our findings suggest that while the HR‐based analysis demonstrated a significantly lower risk in the olanzapine group, this pattern was not consistently observed in the OR‐based analysis. This discrepancy may reflect methodological differences, particularly the use of time‐to‐event data in HR models or the increased variance from limited sample sizes in the OR subgroup analyses.

### Interpretation of HR versus OR analyses

Although even the subgroup analysis of ORs by follow‐up duration (≤24 months vs. >24 months) did not reveal statistically significant differences, the meta‐analysis based on HRs showed a significant difference between olanzapine and clozapine. Given that HRs incorporate time‐to‐event information, this discrepancy raises the possibility that clozapine may be associated with an earlier onset of diabetes, even if cumulative incidence over time remains similar. However, as the OR‐based subgroup analyses did not demonstrate a consistent pattern by follow‐up duration, this interpretation remains inconclusive. The lack of statistical significance may also reflect limited statistical power due to small sample sizes within these subgroups. The moderate heterogeneity was observed in the main OR‐based analysis (*I*² = 34.0%, *p* = 0.15), whereas no heterogeneity was present in the HR‐based analysis (*I*² = 0%). This variation possibly reflects differences in study designs, as the HR analysis was largely based on consistent large cohort datasets, while the OR‐based analysis incorporated a broader mix of study designs, including smaller case‐control studies. However, the apparent homogeneity in the HR analysis should be interpreted with caution given the small number of studies (*n* = 7).

We acknowledge that the patient characteristics between olanzapine and clozapine users are inherently different, which represents an important limitation of this study. Clozapine is typically prescribed for patients with more severe or treatment‐resistant schizophrenia, and it is plausible that such populations may also carry a higher baseline risk of developing diabetes. Nevertheless, it is important—at least in this regard—to highlight the paradox that Japanese regulatory policy permits clozapine, despite its frequent use in these higher risk populations, while prohibiting olanzapine in patients with diabetes.

### Beyond diabetes incidence: Broader implications

The current meta‐analysis focused on the risk of new‐onset diabetes as the primary outcome, because olanzapine and quetiapine are formally contraindicated in patients with diabetes under Japanese regulatory guidelines.^13^, ^45^ However, this outcome represents only one dimension of the broader construct of metabolic safety. Other clinically relevant endpoints—such as changes in HbA1c, insulin requirements, weight gain, and particularly the incidence of DKA—were not included in the quantitative synthesis.

Among these, DKA is especially important due to its acute severity and its potential impact on regulatory decisions regarding drug safety. In Japan, the contraindication of olanzapine and quetiapine in patients with diabetes is partly driven by concerns about several cases of severe DKA, including fatal cases, that occurred within the first year of their approval.^46^ Although several case reports and pharmacovigilance data have linked olanzapine to DKA,^47^ we were unable to include this outcome due to insufficient data of DKA stratified by antipsychotic agent.

Notably, however, a substantial proportion of patients are diagnosed with diabetes at the time of DKA onset, indicating a close epidemiological association between new‐onset diabetes and DKA risk.^48^ Accordingly, the incidence of new‐onset diabetes may, to some extent, serve as a surrogate marker for identifying populations at elevated risk for DKA. Still, given its clinical relevance and regulatory implications, DKA warrants further investigation in future studies.

Unlike previous meta‐analyses, our study prioritized adjusted effect estimates to control for key confounding factors including age, gender, ethnicity, comorbidities, and concomitant medications. This approach enhances the internal validity and interpretability of the results. Our findings suggest that the relative magnitude of risk between olanzapine and clozapine may differ from conventional assumptions. As these results are preliminary evidence based on indirect comparisons of observational studies, the current regulatory classification may warrant cautious reconsideration in future evaluations.

Reclassifying olanzapine as “use with caution,” in line with clozapine, may represent a more proportionate approach under the Japanese regulatory framework. Clinically, when early signs of glucose dysregulation are observed, careful monitoring and timely pharmacological interventions should be implemented, as is common practice in other countries. Moreover, previous studies have demonstrated that quetiapine is associated with a glycemic risk comparable to that of clozapine or olanzapine,^31^ or even lower,^7^, ^34^ suggesting that regulatory reassessment should also be considered for quetiapine.

## LIMITATIONS

Several limitations should be acknowledged. First, most included studies lacked direct comparisons between olanzapine and clozapine, necessitating indirect comparisons using the Bucher method. This approach assumes similarity in study populations and designs across the included studies; however, if this assumption does not hold, bias may be introduced. Second, heterogeneity in outcome definitions, diagnostic criteria, and follow‐up durations may limit generalizability of the findings. Third, although adjusted effect estimates were used, residual confounding likely remains due to the observational design. Several studies did not adequately adjust for key metabolic risk factors, such as baseline glucose levels, body mass index (BMI), or concomitant medications. Fourth, the analysis focused solely on new‐onset diabetes, excluding other metabolic outcomes. Further studies encompassing a broader range of indicators are needed to provide a more comprehensive assessment of metabolic safety. Finally, the small number of studies (HR: *n* = 7; OR: *n* = 9) limits the power to detect publication bias, despite nonsignificant results from Egger's and Begg's tests.

## FUTURE DIRECTIONS

Given the distinct regulatory stance in Japan, future studies should focus on generating robust evidence from Japanese populations. To this end, we are conducting a multicenter observational cohort study, and plan to integrate these data with the existing evidence in an updated meta‐analysis.

## CONCLUSION

Our meta‐analysis, based on adjusted estimates from observational studies, found that olanzapine was associated with a lower or comparable risk of new‐onset diabetes relative to clozapine. However, the overall certainty of evidence remains limited, as most included studies relied on indirect comparisons derived from observational data. These results should therefore be regarded as preliminary evidence. Future direct comparative studies are warranted to provide more robust conclusions. With such accumulated evidence, the current regulatory restrictions on olanzapine (and quetiapine) in Japan could then be reassessed more definitively.

## AUTHOR CONTRIBUTIONS

**Keigo Onda**: Conceptualization; study design; database search; data extraction; statistical analysis; drafting and revision of the manuscript. **Rizal Ichwansyah**: Database search; data extraction; quality assessment; critical revision of the manuscript. **Shuken Boku**: Supervision; interpretation of findings; critical revision of the manuscript. All authors read and approved the final manuscript.

## CONFLICT OF INTEREST STATEMENT

The authors declare no conflicts of interest.

## ETHICS APPROVAL STATEMENT

N/A.

## PATIENT CONSENT STATEMENT

N/A.

## CLINICAL TRIAL REGISTRATION

N/A.

## Supporting information

Supporting Information.

Supporting Information.

Supporting Information.

## Data Availability

All data analyzed in this study were obtained from published articles. Extracted datasets and analytic R scripts used in the meta‐analysis are available from the corresponding author upon reasonable request.
