# Supplementary figures and images for: Risk of diabetes mellitus with olanzapine compared to clozapine: A systematic review and meta‐analysis
Source: PCN Rep. 2025 Oct 14;4(4):e70215. doi: 10.1002/pcn5.70215 (PMC12521611; doi:10.1002/pcn5.70215)

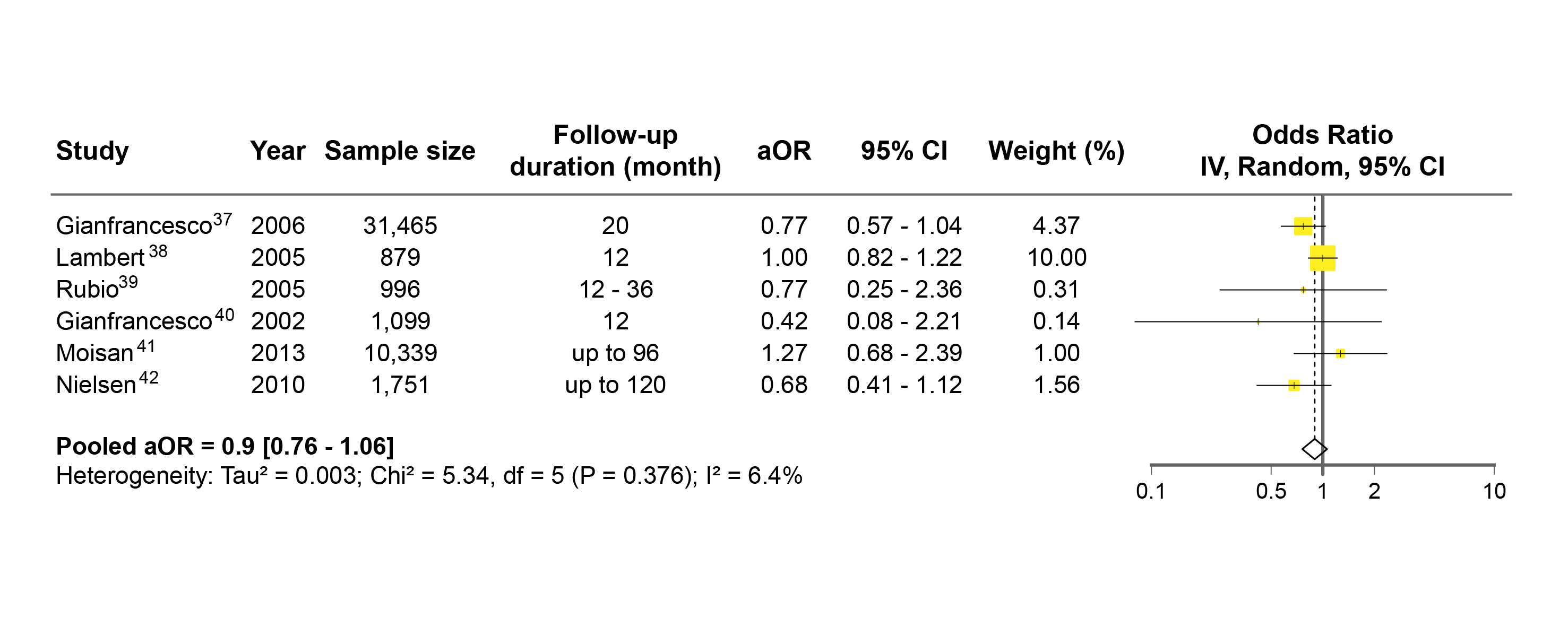

Supplement: Supplementary file 1 — Supporting Information. [file PCN5-4-e70215-s002.png]

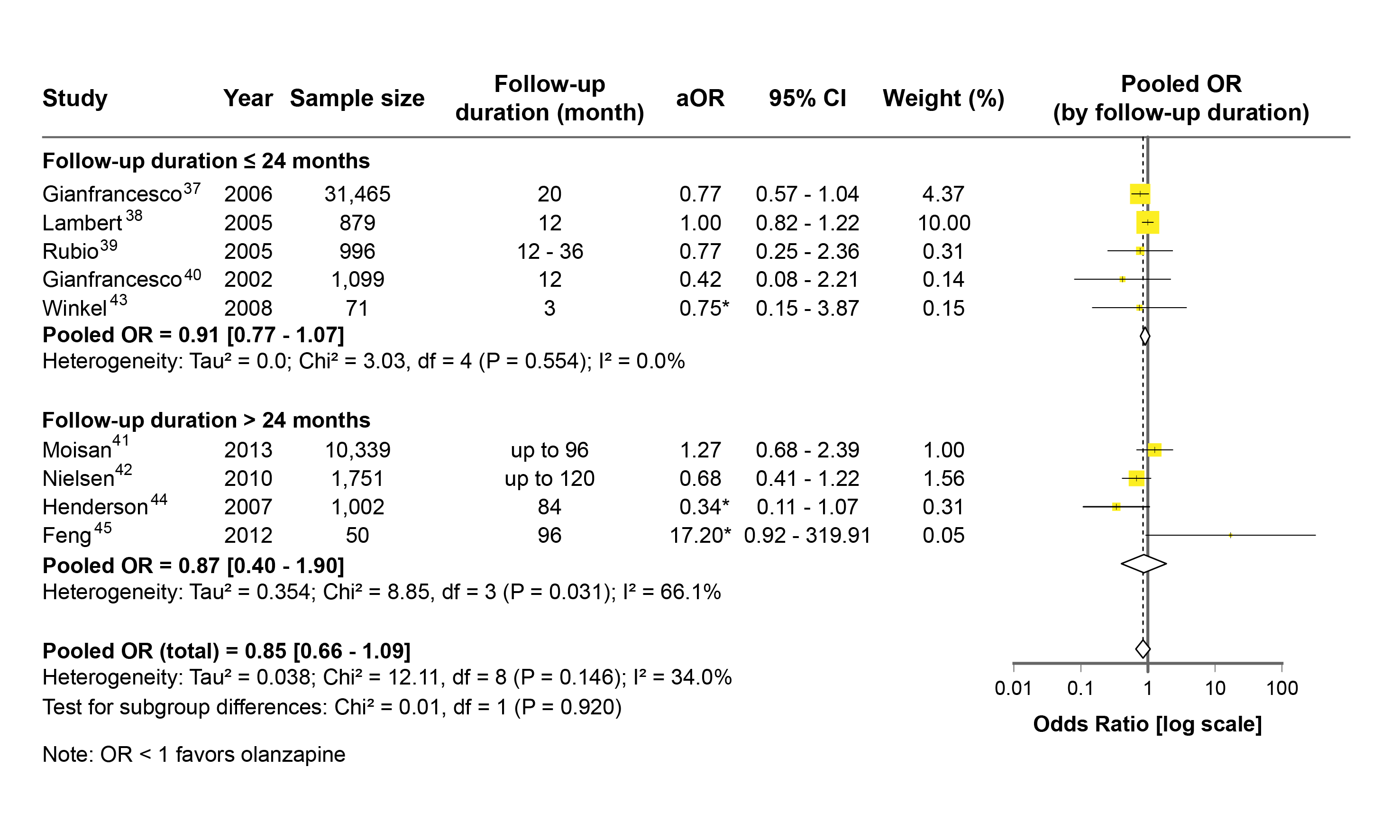

Supplement: Supplementary file 2 — Supporting Information. [file PCN5-4-e70215-s003.png]
