# Supplementary material for: Risk of diabetes mellitus with olanzapine compared to clozapine: A systematic review and meta‐analysis
Source: PCN Rep. 2025 Oct 14;4(4):e70215. doi: 10.1002/pcn5.70215 (PMC12521611; doi:10.1002/pcn5.70215)
Supplement: Supplementary file 3 — Supporting Information. [file PCN5-4-e70215-s001.docx]

Supplementary Table S1. Detailed study characteristics

| ID | Data source | Duration | Analysis method | Covariates included | Comments |
| --- | --- | --- | --- | --- | --- |
| 30 | AdvancePCS prescription claims database | Dec 1998 - Aug 2000 | Cox proportional hazards regression | Age, gender, duration of antipsychotic exposure (dose and time) | Obesity, ethnic origin, and family history were not available in the database and could not be adjusted for. |
| 31 | Kaiser Permanente Health Plan (Northern California) HealthCore Integrated Research Network PharMetrics (covering 73 US health plans) | November 2002 - March 2005 | Cox proportional hazards regression | Age, gender, Index year, Study site, History of antipsychotic use, Exposure to other pharmacotherapy (e.g., beta blockers, corticosteroids, statins, thiazide diuretics, valproate), Obesity (ICD-9: 783.1 or 278.01), Schizophrenia (ICD-9: 295.x, etc.), Bipolar disorder (ICD-9: 296.x, etc.) |  |
| 32 | Veterans Health Administration (VHA) databases | January 1, 1999 - September 30, 2001 | Cox proportional hazards regression | Age, gender, race/ethnicity, marital status, use of potentially diabetogenic medications (beta-blockers, thiazide diuretics, lithium, phenytoin, corticosteroids), and number of glucose metabolic panel tests during follow-up |  |
| 33 | Multistate Medicaid managed care claims database (PharMetrics) | January 1, 1998 - December 31, 2002 | Cox proportional hazards regression | Age, gender, bipolar follow-up duration, use of lithium, anticonvulsants, antidepressants, other concomitant drugs (e.g., corticosteroids, β-blockers), psychiatric comorbidities (e.g., substance abuse), medical comorbidities (e.g., hypertension, weight gain) | Schizophrenia was excluded; only bipolar disorder was included. |
| 34 | MEDSTAT’s MarketScan database (privately insured individuals, including employees, dependents, and early retirees) | Jan 1999 - Oct 2000 | Cox proportional hazards model | Age, gender, stable antipsychotic medication, mental health diagnoses (e.g., schizophrenia, PTSD, depression), clinical comorbidity index |  |
| 35 | Administrative data from the U.S. Department of Veterans Affairs (VA) | June 1999 - September 2000 | Cox proportional hazards models | Age, gender, stable antipsychotic medicaiton, race, income, comorbid mental health diagnoses, levels of service use, VA service-connected disability | The original article reported only totals; olanzapine users were estimated at ~14,000 and clozapine users at ~2,000?2,300. |
| 36 | PharMetrics Patient-Centric Database | September 1995 - September 2001 | Cox proportional hazards model | Age, gender, health plan type, region, calendar year of drug initiation, number of diabetes screening and laboratory tests, psychiatric comorbidities (bipolar disorder and depression), medical comorbidities (hypertension, cardiovascular disease, obesity, impaired glucose tolerance), total duration of therapy, and number of prescriptions for the index medication. |  |
| 37 | Ohio Medicaid program | 1999 - Mar 2003 | Logistic regression | Age, gender, race and ethnicity, type of psychosis, length of observation and treatment, antipsychotic dosage, preexisting excess weight or dyslipidemia, and use of other drugs with potential diabetogenic effects. |  |
| 38 | California Medicaid claims database (Medi-Cal) | January 1995 - September 2000 | Logistic regression | Age, gender, ethnicity, concomitant medications associated with diabetes risk, and antipsychotic exposure |  |
| 39 | the Mental Health Center of Retiro | September 2001 - March 2002 | Logistic regression | Age, gender, BMI (including obesity ?30 kg/m?), smoking status, alcohol consumption, elevated cholesterol, and DSM-IV diagnosis (schizophrenia or schizoaffective disorder). |  |
| 40 | two U.S. health plans (Northeast and Southeast regions) | Jan 1996 - Dec 1997 | Logistic regression | Age, gender, type of health care coverage, observation period, duration and dosage of antipsychotic treatment (in risperidone equivalents), psychiatric diagnosis (schizophrenia, bipolar/manic, major depressive disorder, dementia, or other psychoses), use of other psychotropic drugs, and concurrent use of other antipsychotics. |  |
| 41 | RAMQ (Quebec Health Insurance Board databases), Quebec hospitalization registry | Jan 2000 - Dec 2007 | Logistic regression | Age, gender, obesity, residency area, beneficiary type, use of medications associated with weight gain or diabetes risk, number of different drugs acquired, and use of health services (medical visits and hospitalizations). |  |
| 42 | Danish Central Psychiatric Research Registry, national prescription database | 1997 - 2007 | Logistic regression | Age, gender, use of lipid-lowering and antihypertensive medications, percentage of follow-up time spent as an inpatient, and average antipsychotic defined daily dose (DDD) during follow-up. | Logistic regression analysis was performed to compare the medications used immediately before the onset of diabetes. |
| 43 | University Psychiatric Center, KU Leuven | Nov 2003 - Jan 2007 | Fisher's exact test (independently performed by the authors) | No | As events, sample size, and definitions are clear, the study can be included in the meta-analysis, noting the lack of confounder adjustment. |
| 44 | Research Patient Data Registry (RPDR) (Massachusetts General Hospital) | Jan 1995 - Dec 2001 | Fisher's exact test (independently performed by the authors) | No |  |
| 45 | Psychiatric outpatient clinics in the Stockholm region, Sweden | 2001/2002 - 2009/2010 (8 years) | Chi-square | No | 0-cell corrected; Fisher's p=0.011 |

Extended information on included studies, including data source, study duration, analysis methods, covariates, and additional comments.

Abbreviations: DDD, defined daily dose; BMI, body mass index; PTSD, post-traumatic stress disorder.
